# Successful delivery in a woman with ventricular septal defect complicated with Eisenmenger syndrome: case report

**DOI:** 10.11604/pamj.2022.43.188.32982

**Published:** 2022-12-09

**Authors:** Sara Ait Souabni, El Habib Belhaddad

**Affiliations:** 1 Cadi Ayyad University, Faculty of Medicine and Pharmacy of Marrakesh, Marrakesh, Morrocco

**Keywords:** Eisenmenger syndrome, ventricular septal defect, congenital heart disease, high-risk pregnancy, case report

## Abstract

Eisenmenger syndrome is a dramatic complication of certain heart diseases. It is an absolute contra-indication to pregnancy because it holds a high risk of maternal-fetal mortality. Nowadays, we don´t see much of this condition, because congenital heart defects are diagnosed during childhood, infancy, or prenatally; and even if a pregnancy occurs, it is usually terminated as soon as it is diagnosed. Nevertheless, some women are willing to carry the risks that come with their condition. We report the case of a 26-year-old woman with an undiagnosed interventricular septal wall defect that evolved into Eisenmenger syndrome during her pregnancy. A successful elective C-section was done under epidural anesthesia and close monitoring. When facing this challenging situation, there are no clear guidelines, however, premature and elective delivery is paramount.

## Introduction

Maternal mortality due to cardiac disease is one of the leading causes of maternal death in developed countries [1]. Congenital heart diseases are usually diagnosed since infancy, and women who have it can, with adequate management, have a successful pregnancy. However, some conditions still carry an important mortality risk and until now, remain contra-indications to pregnancy. Among these conditions, untreated left-right communications are one of the most dangerous ones, and pregnancy is usually terminated as soon as it is diagnosed [1,2]. Therefore, the reported cases that discuss pregnancies that are carried out until the third trimester are very limited, and there is no standardized approach when facing this unique situation [2]. In this article, we report the case of an otherwise healthy young primigravida that was carrying an undiagnosed ventricular septal wall defect, complicated by Eisenmenger syndrome. She presented with a rapidly progressing shortness of breath and cyanosis at 33 weeks + 2 days of gestational age. We describe our management and discuss the different therapeutic options.

## Patient and observation

**Patient information**: we report the case of a 26-year-old primigravida, with no past medical history. She presented with stage III dyspnea that was developing for approximately 1 month and was progressively worsening. She was pregnant at 33 weeks + 2 days according to the first-trimester ultrasound.

**Clinical findings**: on clinical examination, she was cyanotic, tachycardic (100 bpm) with a slightly elevated respiratory rate (22 cycles/min) and normal blood pressure (110/75mmHg). Saturation was at 82% in open-air and there was no visible retraction of the respiratory muscles. There was a loud P2 on cardiac auscultation, but no signs of right or left heart failure. The pulmonary examination was unremarkable.

**Figure 1:**
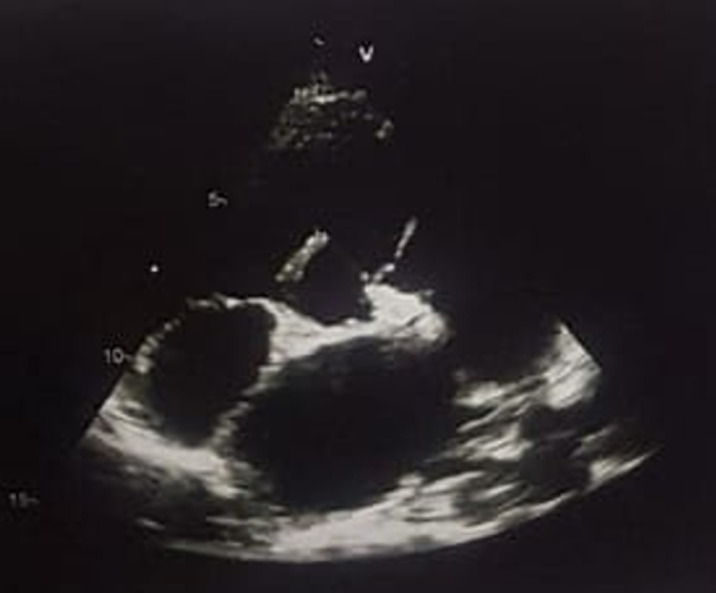
transthoracic echocardiography showing ventricular wall defect with dilatation of the four cardiac cavities

**Figure 2:**
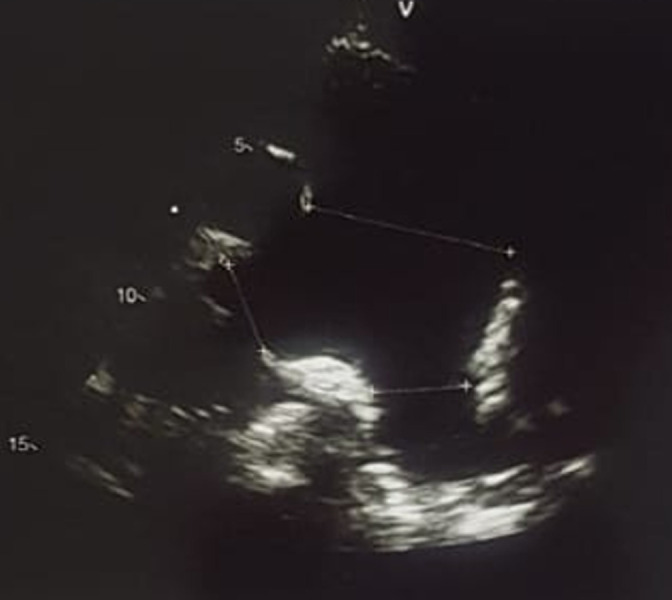
massive dilatation of the main pulmonary artery measuring 55mm

**Diagnostic assessment**: an ECG was performed and showed sinus tachycardia with electrical left ventricular hypertrophy and auricular hypertrophy. The cardiac ultrasound showed a large sub-aortic interventricular communication measuring 16mm with bidirectional shunt predominantly right-to-left shunt, dilated right cavities and hypertrophied left ventricle and left auricle (Figure 1) with preserved function, severe pulmonary hypertension with aneurysmal pulmonary artery measuring 55mm and PAPS = 122mmHg (Figure 2). On the obstetrical side, the woman was at 33 weeks and 2 days of gestational age. She was not in labor. The ultrasound revealed a progressive mono-fetal pregnancy with breech presentation, with normal amniotic fluid quantity, fundal placenta, and estimated weight at 1931g ± 282g.

**Therapeutic interventions**: we hospitalized her in the maternal intensive care unit and started her on diuretics, anticoagulation, and oxygen. Betamethasone for fetal pulmonary maturity was also administered. We performed an elective C-section under epidural anesthesia the next day. We did not perform a tubal ligation due to the couple´s refusal.

**Follow-up and outcome of interventions**: the procedure went without incident, and there was minimal blood loss. No perioperative or postoperative complications were noted. The newborn was a male, 1kg900g weighing baby, with an APGAR score at 8/10 in 0 min and 10/10^th^ at 5 minutes. He was hospitalized in a neonatal intensive care unit for further care.

**Informed consent**: the patient reported her full consent to publish her case.

## Discussion

Eisenmenger syndrome (ES) is a dramatic complication of untreated left-right communication, that was first described by Viktor Eisenmenger in 1897 [2]. The increased pulmonary flow results in the progressive alteration of the pulmonary arterioles, which leads to the increased pulmonary resistance, that eventually becomes greater than systemic resistance, resulting in a reversal of the shunt with cyanosis. The fall in systemic vascular resistance (SVR) during pregnancy increases right to left shunting, originating in cardiac decompensation and low output [1].

When it is diagnosed, patients should be sensitized to the dangers that pregnancy carries, and advised about the use of effective contraceptive methods [3]. If pregnancy occurs anyway, pregnancy termination is recommended. It can be done by dilatation and evacuation, or by medication; ideally before 10 weeks´ gestation [4]. If the patient chooses to preserve the fetus, she should be closely evaluated, with frequent monitoring in a tertiary care center [5]. Early admission may be required [4]. The medical management consists of digitalis, diuretics, vasodilators, anticoagulants, and oxygen [6]. Oxygen should be given during delivery because it decreases right-to-left shunt by decreasing blood flow which accordingly increases O_2_ saturation. Diuretics are most beneficial when there is severe right heart failure. However, caution should be taken when it is associated with digitalis, so as not to have any toxicity [6]. Although endothelin-receptor antagonists and phosphodiesterase 5 are medications that have proven to be effective in pulmonary hypertension, they are also teratogenic. Therefore, they are not used during pregnancy, but they can be utilized in the post-partum period [3]. The administration of inhaled Nitric oxide during labor has proven to attenuate pulmonary arterial pressure, so it is now recommended [7].

Regarding anticoagulation in ES patients, it should be used with caution, because of the existence of thrombocytopenia and hemoptysis risks, that add themselves to the risk of postpartum hemorrhage [1]. That is why in every case, pros and cons should carefully be assessed [7]. But generally speaking, low-dose heparin during bed-rest is reasonable [5]. In all cases, early delivery is necessary because the hemodynamic condition of the mother can quickly deteriorate [8]. Delivery before 32 weeks´ gestation seems to be beneficial [9], and the use of betamethasone for fetal pulmonary maturity can be done safely [6]. However, the mode of delivery is not consensual yet. The options that present themselves are elective cesarian section or planned vaginal delivery. The European cardiac society recommends performing an elective C-section for patients with pulmonary hypertension, but they did not provide clear guidelines for Eisenmenger syndrome per se [8]. Many authors are in favor of this option because it prevents emergency C-section that holds, in itself an increased risk of complications that are mainly due to less careful preparation [9]. Also, the auto-transfusion phenomenon that occurs during each uterine contraction is an additional risk to consider [5]. On the other hand, some argue that vaginal delivery is best because it is better tolerated in cardiac conditions, and there is a lower risk of general complications, especially those related to blood loss and thrombotic problems [9]. When planned vaginal delivery is chosen, there should be short labor, with adequate anesthesia and avoidance of arduous expulsive efforts [4]. This could be a possible indication for vacuum or forceps-assisted delivery [4]. The best anesthetic option for ES during labor or delivery is epidural because it provides the best hemodynamic stability compared to other alternatives [8]. Finally, some recent articles introduced the utility of preparing an ECMO during delivery, and having it available as a standby option in case of complications, because it could be, in some cases, a life-saving procedure [8].

**Strengths and limitations of our case report**: our case gives a good description of the symptoms, the clinical findings, and the management of Eisenmenger syndrome during the third trimester of pregnancy, which adds to the scarcity of data about the subject. However, studies with a bigger sample are vital to be able to draw better conclusions.

## Conclusion

There is limited information regarding the management of Eisenmenger syndrome during the third trimester of pregnancy. The management in a third-level hospital with a multidisciplinary team is always necessary. Elective and premature delivery, whether it is by vaginal delivery or cesarian section seems to improve maternal-fetal outcomes. Vasodilators that are used in pulmonary hypertension are teratogenic, and can only be used after delivery. The anesthesia during labor and delivery is best and more safely obtained via epidural. The availability of an ECMO nearby can sometimes be life-saving.
